# Using Co-Design to Develop a Health Literacy Intervention with Socially Disadvantaged Adolescents

**DOI:** 10.3390/ijerph19094965

**Published:** 2022-04-19

**Authors:** Hannah R. Goss, Craig Smith, Laura Hickey, Johann Issartel, Janis Morrissey, Celine Murrin, Ailbhe Spillane, Sarahjane Belton

**Affiliations:** 1 School of Health and Human Performance, Dublin City University, D09 NA55 Dublin, Ireland; craig.smith57@mail.dcu.ie (C.S.); johann.issartel@dcu.ie (J.I.); sarahjane.belton@dcu.ie (S.B.); 2 The Irish Heart Foundation, D06 C780 Dublin, Ireland; lhickey@irishheart.ie (L.H.); jmorrissey@irishheart.ie (J.M.); 3 School of Public Health Physiotherapy and Sports Science, University College Dublin, D04 V1W8 Dublin, Ireland; celine.murrin@ucd.ie (C.M.); ailbhespillane@gmail.com (A.S.)

**Keywords:** schools, health education, wellbeing, participatory methods, inequalities

## Abstract

The aim of this study was to initiate a co-design process with adolescents to inform the development of a targeted health literacy intervention for implementation in designated socioeconomically disadvantaged post-primary schools in Ireland. Purposely developed vignettes were explored in a series of eight workshops that were conducted separately with staff (*n* = 26) and students (*n* = 33) across four schools. Data was analysed using content analysis. A number of key health topics were identified as important and influential for the participants in this context: food choices, mental health and wellbeing, physical activity and sedentary behaviour, sleep and substance misuse. Participants also suggested many health-related capacity building actions. Participants recognized that many of these health topics and capacity building actions were intertwined and also highlighted that some of these actions may be more feasible and/or impactful than others. For example, students and school staff both indicated the need to use relevant, applied and engaging approaches to improve health literacy and subsequent health behaviour. The co-design process adopted empowered stakeholders to actively engage in the design and development of future intervention strategies, which may increase the likelihood of acceptability, effectiveness and sustainability of the resulting intervention.

## 1. Introduction

Adolescence is a critical period of transition in the life course where health values, knowledge, skills and behaviours are developed and established [1]. Research has highlighted that risk behaviours for non-communicable diseases (NCD), the leading cause of premature adult deaths, are often initiated in adolescence [1,2,3]. There is consistent evidence of a persistent and potentially widening socioeconomic gap in health indicators in adolescents [4]. There is also evidence of health disparities in youth in Ireland across a number of different health indicators including: Body Mass Index (BMI) [5,6]; physical activity [7]; health-related fitness [6]; mental health [8]; smoking [9]; dietary behaviour [10]; sleep [11]; and alcohol and substance misuse [12]. Irish research has also indicated that children attending disadvantaged schools are at a higher risk of being overweight or having obesity than children in non-disadvantaged schools [6].

Health literacy has been positioned as a concept that may be of use in understanding these health disparities [13]. Health literacy is the ability of an individual to find, understand, appraise, remember and apply information to promote and maintain good health and wellbeing [14,15]. Research has demonstrated the concept of health literacy to be an independent factor for: explaining disparities in health [16,17,18]; being a mediator for health behaviours [19,20,21]; being related to positive outcomes [22,23,24]; and crucially being a modifiable factor that can be changed by educational means [25,26,27,28]. Despite the potential impact of adopting a salutogenic approach to improving health outcomes by targeting health literacy in young people, research focusing specifically on health literacy in non-clinical youth populations, and specifically young people from social disadvantage, is under researched in comparison to adult populations [29].

The Irish Heart Foundation (IHF) Schools Health Literacy project, which is a registered World Health Organisation (WHO) National Health Literacy Demonstration Project [30], is focused on co-producing a school-based health literacy intervention with and for young people aged 12–16 years old from socioeconomically disadvantaged communities. The timing of this project has also coincided with a renewed focus on wellbeing within the Irish school system [31] and the publication of Irish research that has stressed the importance of positioning young people at the centre of the learning experience in curriculum development [32]. Participatory approaches present the opportunity to do research “with” rather than “on” participants. While citizen participation is not new [33], it continues to face challenges such as tokenism, limited impact and unsustainability [34]. When working with young people in particular, Tisdall (2017) cited further issues: the lack of reciprocal information-sharing and dialogue, the lack of impact on decision making and perceptions of vulnerability [35]. However, there is huge value in centring on the young person’s voice and learning about their life experience. Although there are varying degrees of engagement in participatory research [36], co-design is commonly understood to present an opportunity for meaningful end-user engagement throughout the research process [30]. In the context of schools and young people, co-design refers to inviting the school community and external stakeholders to participate in a design or problem-solving process, and, crucially, all ideas and knowledge are equally appreciated throughout the process [37]. A recent systematic review of school based co-design approaches identified several consistent themes across the cited interventions, including: capacity-building, structure and recruitment, group development, problem setting, problem solving, adoption, implementation and evaluation [37]. To our knowledge, this process has not been undertaken in a non-clinical socially disadvantaged youth population. As a result, this study aimed to explore the health needs, practices and ideas of students and staff in low socioeconomic schools in Ireland through initial co-design workshops to develop a future health literacy intervention.

## 2. Materials and Methods

### 2.1. Study Design

The present study is a descriptive cross-sectional study based on qualitative research methodology. Ethical approval for this study was granted by the institutional research ethics committee [DCUREC/2019/053].

The Ophelia (Optimising Health Literacy and Access) process has been developed to specifically guide the co-design of health literacy interventions [38,39]. Ophelia draws upon a set of well-established intervention methodologies and is a flexible guide to help identify local health strengths, needs and solutions [39]. Adopting a realist approach, it focuses on contexts, mechanisms and outcomes and involves iterative and low-quality improvement cycles before large scale implementation [38]. In particular, this paper focuses on Steps 2 and 3 of Ophelia and details the process and findings regarding the needs assessment and idea generation in this context. Details regarding Step 1 of this project have been published elsewhere [40].

### 2.2. Participants

Four post-primary schools (*n* = 3 mixed-gender schools, *n* = 1 all-male school), who were previously purposively recruited to participate in the larger IHF WHO Health Literacy Demonstration project, were involved in this study (see Table 1) One all-girls school previously involved in this project declined to participate in the current study due to lack of time. These schools were recruited as they were all part of the Irish Department of Education’s “Delivering Equality of Opportunities in Schools” (DEIS) action plan, which aims to address educational disadvantage [41]. The authorship team comprised of researchers and practitioners with extensive experience working and researching in this context and all resources were piloted with teachers and students from similar school environments prior to their use in the study. Students were recruited through the school project contact as part of the ongoing Healthy Literacy Demonstration Project. There was no strict inclusion or exclusion criteria, but school contacts were asked to identify approximately eight students with a range of diverse backgrounds and interests across the three years of Junior Cycle education (first to third year; age 12–15 years), who would be willing to join the workshops. The project was explained to the students by the school project contact and they (and their parents) were given a plain language statement and consent/assent forms to complete. Recruitment of school staff consisted of the school project contact explaining the project to their staff and identifying and inviting interested school staff to participate. Parental consent (for students), participant assent and gatekeeper (i.e., school principal or school management) consent were obtained prior to data collection. The range of school staff that participated varied slightly by school, but in general included teachers from a range of subject areas (including physical education, science, SPHE (Social, Personal and Health Education), home economics, maths) as well as Home School Liaison Officers, School Counsellors and School Chaplains.

### 2.3. Procedures

The two co-design workshops per school (one student and one teacher) were conducted separately throughout winter 2019–2020 (prior to the COVID-19 pandemic). All co-design workshops followed Ophelia guidance [38,39] and were conducted with three researchers present acting as the (i) facilitator (HG), (ii) co-facilitator/scribe (SjB) and (iii) a critical observer (LH). The roles of facilitator/scribe were fulfilled by experienced researchers who had previous experience in qualitative research and working in schools. The role of critical observer was fulfilled by a team member with significant experience working with schools, teachers and children; they were introduced to the group at the start of the session but sat at the back of the room as activities were taking place. Typically, co-design workshops were conducted in a classroom or another quiet space (e.g., staff room) within the school. The space used had a projector to enable the co-facilitator to keep note of all ideas and present them back to the group in real time.

The research team developed and followed a structured protocol. At the start of each workshop, as an icebreaker participants were presented with a range of images and asked to individually select two images that they felt represented “heath issues in your school”. Participants were then invited to introduce themselves to the group and share a brief explanation of why they chose their images. Following this activity, the first vignette was introduced to the group. A series of nine vignettes were created as part of the wider project; these vignettes and details of their development are available elsewhere [40]. In short, these vignettes were evidenced-based fictional case studies depicting characters with typical health literacy profiles of adolescents in this context. This included narrative regarding understanding, awareness, strengths, needs and issues in relation to health literacy and health in this context. Crucially, these vignettes were derived from mixed method research previously conducted in the same participating schools [40]. Vignettes were on average 300 words long and used words and phrases offered by the students themselves in previous focus groups and validation checks. They provided authentic and tangible descriptions of the health topics and health literacy profiles of adolescents in these specific schools [40]. Workshops lasted a maximum of two hours. As a result, student co-design workshops discussed three vignettes, whilst teacher workshops discussed two vignettes each. When allocating the specific vignettes for discussion in each workshop, the research team considered participant demographics (e.g., male vignettes presented in the male school), which ensured a range of health literacy profiles and health topics were represented in the vignettes chosen. Additionally, all vignettes were presented at least once to students and teachers across the eight workshops, in line with previous research [42]. For each vignette discussion, each participant was provided with two A4 printed pages: a written copy of the vignette and an associated storyboard. The project team incorporated the use of storyboards in the co-design workshops to promote accessibility and engagement with the vignettes. These story boards each compromised six key quotes from the vignette; each quote was accompanied by a corresponding image. Quotes and images were selected by the project team, who were mindful of best practice guidelines around stigmatising graphics. Large posters of the vignette storyboards were also displayed in view of the participants. The vignette was read aloud by the facilitator to ensure all participants were familiar with the character.

A series of semi-structured question guides accompanied each vignette discussion, with appropriate wording selected for student and teacher workshops, respectively. Four key areas were covered: (i) is this vignette character familiar? (ii) what sort of issues is this person facing? (iii) what strategies could help a person like this? (iv) what could be done if there are lots of people like this in your school? Question prompts specific to the vignette’s character were also employed as needed. Where appropriate, the facilitator referred to text and images to stimulate conversation.

Insights and ideas were recorded by the co-facilitator and they were displayed in real time on a large screen for participants to see. These notes were presented back to participants iteratively as the workshop progressed to ensure accuracy and understanding as part of initial respondent validation. Vignettes were discussed until saturation, which was deemed to be reached when participants had no additional comments to the co-facilitator’s notes, which were frequently summarised and reflected back to participants. This enabled participants to check for accuracy and resonance with their experience and thus demonstrate the credibility and trustworthiness of findings. A further vignette was then presented following the same protocol, as time allowed.

With approximately 15 min to go, the workshop was brought to a close by asking participants “which are the three most important ideas that you have come up with?” and “what are the three easiest ideas to implement in your school?” from across all the vignettes, before finally asking participants if there was anything more they wished to add. All workshops were audio-recorded using a digital dictaphone to ensure no ideas were missed.

### 2.4. Analysis

The research team conducted content analysis of the discussion that took place in each workshop. Firstly, student and school staff perceptions were identified within each workshop by the co-facilitator, which were presented back to participants towards the end of the workshop. These points were interpreted, critiqued, additions made and agreed upon by participants themselves to promote authenticity of findings [43]. After completion of each workshop, the critical observer also offered insight to the research team as to any ideas or inferences that may have been missed. The recordings were subsequently listened to and cross referenced with the notes from the co-facilitator and critical observer to abstract the key points from each workshop. After completion of all workshops, researchers (H.G., C.S., S.B. and J.I.) synthesized findings through reflexive discussion into common concepts [44,45]. The use of multiple researchers throughout this reflexive process is common in other projects adopting the Ophelia framework [46,47,48,49]. In line with the Ophelia framework [38,39] and the aims of the study, analysis of the insight is focused on the development of useful knowledge. In this case, concepts are directly related to the development of health literacy in socioeconomically disadvantaged Irish post-primary schools.

## 3. Results

The purpose of this study was to facilitate initial co-design workshops with schools of designated low socioeconomic status with a view to exploring young peoples’ health needs, practices and ideas to inform the co-design of a health literacy intervention. Participants indicated that five key health topics were especially relevant in this context (in no particular order): food choices, mental health and wellbeing, physical activity and sedentary behaviour, sleep and substance misuse (see Table 2). Capacity building actions, i.e., tangible and pragmatic solutions, were suggested by participants in relation to the health literacy needs of individuals and populations in this context both in relation to specific health topics and in relation to health more widely; as a result, these are also included in Table 2 and throughout the results narrative.

### 3.1. Food Choices

Food and nutrition were frequently spoken about in relation to “being healthy”. Teachers spoke of the importance of students’ autonomy “…but if they had ownership of what they want to do” in relation to their food choices, food education and cooking activities. Teachers also spoke of a need to “convince staff of the links between improved health and nutrition, and improved health behaviour”. Although students and teachers spoke of how they already learned about how to keep healthy and about the food pyramid, across the schools cooking classes were consistently suggested, not only for the students but also for families and the wider community “so you’re (the students and their families) not depending on takeout’s”. Critically, none of the schools had suitable facilities to provide such classes to significant numbers.

As part of a school’s DEIS status, schools are provided with funding towards the provision of food services for disadvantaged students. In one participating school, where there was not enough space or time to provide seats for all students to sit and eat lunch, teachers spoke of how the food had to be “eaten on the move”. Students across the workshops suggested that to improve food choice and quality, they could be involved in future decisions regarding lunchtimes through a simple survey. One school was in fact already engaging in such a process of change:

“We have a committee, and we’ll be changing what we have (on offer at lunchtime) to give other options.”

In relation to food choices, students gave an example of energy drinks, which were banned in their school. Despite the ban, students spoke of how they were still commonly consumed by students due to their low price and availability in the local shops. Participants suggested that selling such drinks to under 18s should be banned. Furthermore, teachers spoke of the need for more inspiring education around healthy eating:

“young people should be given the opportunity to actively participate to become motivated to make change”.

### 3.2. Mental Health and Wellbeing

Across the schools, mental health was one of the first health topics recognised when discussing the vignettes. Each of the schools, as part of their DEIS status, have access to a dedicated guidance counsellor. In the all-boys school, it was commented that the counsellor was “really calm, she’s very open to your feelings”, but sometimes people “didn’t feel like going in” as there was a stigma around getting support. In other schools, some students were unaware of the counselling services available to them—“no one in our school talks about it”—and it was recommended by the students that these services could be better signposted, rather than relying on the students themselves to seek out support. Furthermore, in relation to mental health and wellbeing, many participants recognised the impact of social media. In the current study, one teacher spoke of the impact of social media on young people’s motivation:

“This generation that has been born to accept technology. And this is the first time we are seeing real problems of it in that they expect everything to be instant success. In social media you see perfection in bodies, all these designer clothes, loads of things. (Social media) Doesn’t provide knowledge and it brings their mood down, lack of motivation. It’s not reality and they have to understand that”.

Students in the all-male school were able to speak of a strategy already in place:

“We do debating in class… we have a club in school… it gets you more objective to see what is true, what is fake. Especially important now with social media, and fake news”.

Notably, although mental health and wellbeing was cited across all workshops as an important health topic for this population, suggestions for mental health-related capacity building actions from school staff were limited.

In a discussion regarding one of the vignettes, a female participant considered the character to be “addicted to her phone” and linked this to social media use and issues with confidence. It was identified that although there was a no phone policy in the school that these participants attended, this often did not prevent the students from being on their phones in class and participants reported spending a lot of time on their phones outside of school. The same participants spoke of checking their screen time, indicating they were aware of the impact this may have on their health, but they did not believe this awareness of duration had any impact on actually reducing their screen time.

### 3.3. Physical Activity and Sedentary Behaviour

There was also discussion around being physically active outdoors in their local area, with participants speaking of safety concerns playing in their local environment:

“Your estate, like playing around your estate. Especially the younger kids, like if anything happens to you, your house is only right around the corner… like in case of a stranger, or something like that”.

Participants felt unsafe when going to local parks. When prompted by the facilitator for potential solutions to overcome this barrier:

“There’s not much you can do to be honest… Like how are you going to stop? You can only hope for it”.

Other students went on to suggest free (or at least affordable) access to safe sporting facilities in their local area and were also suggesting the provision of non-competitive sport in school. Both school staff and students spoke of the need to provide inclusive activities that catered for all in a range of modes and not just competitive sports teams that already existed in all schools. The need for partnerships with existing schemes was also suggested by both school staff and students. Whilst partnerships with specific organisations were suggested in each of the workshops, each school operates in its own specific context that relates to its particular circumstances and characteristics and, as a result, not all of these suggestions were common across the four participating schools.

Particularly for females, friends ceasing playing sport was commonly cited as a reason for them to drop out in this age bracket. Crucially, this discussion demonstrated that young people need further support to make changes to their health environments, not just their own behaviour. When discussing communities across the schools, many participants spoke of community partnership approaches. This included discussion around existing programmes and organisations, particularly sports clubs, and the need for any future intervention to collaborate with these partnerships.

### 3.4. Sleep

Sleep was seen as a key topic by many participants as it was often linked to other topics such as food choices (i.e., energy drinks), sedentary behaviour (i.e., computer gaming) and wellbeing (i.e., impact on attendance and schoolwork). In a discussion regarding one of the vignettes, students considered the character to be “addicted to her phone” and linked this to social media use and issues with confidence. Student participants in a separate school also spoke of potential solutions to limit screen time:

“Like my friend… he has one of the screen timers on his Xbox. He may be on it for an hour and then he has to go off”.

Findings presented by participants across workshops indicated that although they were able to identify the associated negative health outcomes, they were not aware of any strategy used successfully to decrease screen time in this age group; the consequence of poor sleep was a common problem for many in this age group.

### 3.5. Substance Misuse

Substance misuse was spoken of most commonly in relation to vaping, alcohol and cigarettes, with other drug use being discussed in relation to older age groups, and a potential problem for future health. The impact of peer pressure was also frequently mentioned in relation to smoking (including vaping), alcohol and substance misuse. Substance misuse also came to the fore when discussing the older vignettes with students. Within the staff workshops, many teachers admitted gaps in their own knowledge. Specifically, teachers requested “evidence-based education on the health risks of vaping” as they themselves could not navigate the misinformation around this newer health topic.

## 4. Discussion

The purpose of this study was to detail the process and findings of initial co-design workshops with schools of designated low socioeconomic status, enabling stakeholders to highlight health needs of adolescents and potential implementable solutions. Five key health topics (pre COVID-19) were identified: food choices, mental health and wellbeing, physical activity and sedentary behaviour, sleep and substance misuse. The capacity building actions offered by participants may be considered as potential intervention strategies that could be applied across the health topics identified in this context. Specifically, findings from this study, both health topics and capacity building actions, will directly inform the development of health literacy intervention for socioeconomically disadvantaged Irish post-primary schools as part of an ongoing wider project following the Ophelia framework [38,39].

### 4.1. The Individual

Across different co-design workshops it became clear that although young people may be told about health topics, this information was often not valued by young people and therefore did not translate into behaviour change. This is a crucial consideration, given that being “outcomes focused” is the first guiding principle of the Ophelia framework [38]. Motivation regarding the acquisition of health information has been found to be the most important independent variable when exploring factors associated with children’s subjective health literacy [50] and this was echoed by the participants in this study. This implies that the transmission of knowledge is perhaps not enough without a consideration of whether students want to learn and what they want to learn about [16]. Learning activities, which are carried out through interactive tasks and are focused on context specific learning, may improve adolescents’ ability to obtain, understand and critically assess information [30], ultimately leading to improved health literacy and potentially positive health behaviour change [51]. Students gave suggestions of specific activities such as: structured debates, role play, goal setting and developing a variety of coping strategies. Students also spoke of previous self-directed activity that had already been implemented within their schools. For example, student participants across all schools spoke of poor food quality and in one school students spoke of advocating for the changing of food providers.

As well as potentially improving motivation and self-efficacy, self-directed activities such as this can enable young people to experience the link between health literacy and their everyday lives [52]. It is important, however, that such activities should value young people as competent social actors and critical citizens yet crucially protect them from victim blaming and an unrealistic amount of responsibility [53]. School staff also felt strongly that content and activities should be relevant, engaging and applied. Staff from one particular school spoke of how the students had recently been involved in a successful project to reduce plastic waste and had noted the positive changes in student motivation and engagement. These findings reinforce the potential of transformative and constructivist pedagogy as a means to empower students to critically examine their beliefs, values and knowledge with the goal of developing a reflective knowledge base that is ultimately action orientated [28,54]. This is often achieved through student-centred inquiry-based reflective pedagogies that encourage students to be reflective and critical thinkers (seen as key health literacy capabilities [55,56,57]) who are able to contribute meaningfully as members of local and global communities [58].

As discussed previously, there is consistent evidence of the impact of food choices, mental health and wellbeing, physical activity and sedentary behaviour, sleep and substance misuse on the lives of young people in Ireland [5,6,7,8,9,10,11,12]. This study highlights the awareness of the young people themselves of these impacts. The research team also acknowledges that many of these concepts identified by participants are interrelated. In a pragmatic and actionable approach, we believe this crossover should be welcomed. What is more, many of the insights offered align with the existing WHO Health Promoting Schools (HPS) framework that presents an eco-holistic multicomponent approach to creating school environments conducive to health and health behaviours [59]. Despite Ireland being one of the first members to join the European Network of Health Promoting Schools (ENHPS) at its inception in 1992, the awareness and engagement with the strategy and award by schools nationally has been mixed [60]. While definitions vary, some key areas for action in a HPS framework are identified as the school curriculum, environment and communities [59,61]. Throughout this study, specific strategies in relation to these themes were suggested by participants. Health literacy is not only about individual competencies and behaviours and both students and staff spoke of solutions that would require wider change.

### 4.2. The School

Whilst some of these strategies were spoken of in relation to specific health topics, there were also suggestions relevant across the health topics identified. Many ideas and suggestions presented by the students and teachers in the co-design workshops point to the value of school and, more specifically the school curriculum, as an ideal vehicle to help challenge and address the health topics discussed. Both the school staff and students strongly proposed focusing on younger age groups, in particular first years (12–13-year-olds). Whilst this could be seen as at odds with a “whole school approach”, teachers also felt the first-year curriculum and lack of state-level exams meant there was less pressure on timetabling and resources. Students also spoke of targeting first years as a priority as students felt this age group had not already developed “bad habits”.

In relation to school curriculum, pedagogical approaches and teacher professional development were frequently cited, particularly within the school staff co-design workshops. These participants also spoke of limited training in relation to health, with many early career teachers being timetabled to teach SPHE (Social Physical and Health Education), which was outside of their subject expertise. When asked from whom they wanted to learn about health information, students from one school spoke of a particular teacher who they felt was a good health role model, but more importantly that teacher made them feel comfortable and supported in their lessons. These students also discussed how teachers could go on courses to learn how to lead health-based lessons. In the current study, it is notable that despite mental health and wellbeing being identified as a key health topic, the staff participants did not propose any initiatives in this regard, which perhaps indicates the need to train staff to address mental health issues.

In the Irish context, recent curriculum reform may be timely to present an opportunity to integrate health literacy within Junior Cycle education. A new Wellbeing Framework at Junior Cycle was introduced in 2016 in Irish post-primary schools, with the view to provide each school with the autonomy to design their own programme drawing on a number of curriculum components to reflect the specific needs and resources of the school [31]. In Australia, similar curriculum reform in health and physical education explicitly identified health literacy as one of five key underpinning propositions [62]. Recent research has suggested that adopting such an approach can facilitate the capacity of schools and schooling to make a realistic educative contribution to the health, physical activity and wellbeing of young people [50,55]. When talking about the health needs and potential strategies to overcome these issues, teachers in the current study offered the idea of a short course (see Table 2). This is consistent with changes in national educational policy as advocated by the Irish National Council for Curriculum and Assessment (NCCA) and the Junior Cycle for Teachers (JCT) for starting to integrate the area of Wellbeing within their schools [31]. Although the curriculum changes at Junior Cycle provided the opportunity for schools to take control of content and focus within their curriculum, with minimum hours for wellbeing in Junior Cycle stipulated the teachers in this study expressed a need for support and resources to help align their curricula with the Wellbeing framework whilst reflecting their school’s context [31]. As indicated by the above quote, teachers recognise the need for such a curriculum, but felt they needed support in developing and implementing it in practice. When students were asked from whom they wanted to learn about health information, students from one school spoke of a particular teacher who they felt was a good role model, but more importantly the teacher made them feel comfortable and supported in these lessons. These students also discussed how teachers could go on courses to learn how to lead health-based lessons. This reiterates the importance of empowering teachers to feel confident, competent and comfortable in not only what they are teaching but also the way in which they teach [63]. Teachers should be supported in a strategic and ongoing manner; this may include continuing professional development, resources and mentoring [62]. Further co-design with teachers is necessary to identify how the teachers participating in this project would prefer to receive this support, with particular consideration given to engaging a wide range of representative teachers. Students in the current study recognised lack of sleep and often-associated consumption of energy drinks as a common issue for many young people. This issue was targeted by an existing strategy in one of the schools, the banning of energy drinks. Although this policy was enforced by teachers in school, participants spoke of how students just bought these before or after school. The students recognised that the school may not be able to solve all the challenges and recognised the need for national policy. The participants themselves have recognised that policy is often necessary to support their health behaviours or to change their environment. Improved health literacy can support the development of empowered and engaged citizens.

### 4.3. The Community

Health literacy interventions targeting school-aged children should also link to the wider socio-ecological school environment, this includes organisations, charities and clubs as well as their extended families within the community. In a systematic review of co-produced school-based health interventions, reaching and involving families was acknowledged as being highly challenging [37]. Nevertheless, parental health orientation, self-efficacy and motivation are factors significantly associated with health literacy in school-aged children [16]. Insights from the current study highlighted the changing role of the parents of adolescents, with some students suggesting the parent should take responsibility for the health choices of their children. The changing parental dynamic during adolescence and the extent to which a school-based intervention can influence family health is a challenge. Nevertheless, existing science and health literacy interventions have seen this as an opportunity, whereby learning experiences can empower young people to communicate with their family, peers and community about their learning, questions and actions [28].

As well as giving insight into the barriers and challenges faced by the most vulnerable families in this study, the HSL officers also spoke of previous initiatives to engage families in the school community. In one school, a HSL officer recognised that her ability to relate to the most vulnerable students was in part because she grew up and still lived in the local area. This exemplifies the need to be “driven by local wisdom” [39]. Both teachers and students spoke of the possibility of using external speakers, from the local area or to whom the young people could relate, as positive role models. These speakers may be able to communicate and engage with the students in a different way than their class teachers.

Working with these schools has the potential to develop meaningful relationships and meaningful change. Although implementation of this co-design process is multifaceted, findings from these initial workshops have identified a number of interrelated concepts that can be actioned to facilitate and sustain a whole school culture that values health and wellbeing. As a result, to date, the steps conducted in this project following the Ophelia process have been invaluable in empowering these school communities to identify and start to co-create solutions to improve access, equity and outcomes by addressing health literacy needs [38,39].

### 4.4. Strengths and Limitations

The Ophelia approach has been used in a number of settings and has been shown to be an effective and flexible guide to help identify health literacy challenges and develop and implement locally appropriate solutions. However, in peer-reviewed published material, the methodology of these projects is often reported at the macro level, detailing broadly all steps in the process. As a result, at the micro level, the methodology employed at each step can be unclear to those less familiar with the approach. This can be advantageous in some ways, allowing others to be flexible and innovative in the methodological procedures they undertake when conducting co-design needs analysis with their specific contexts. On the other hand, it can leave questions as to what co-design actually looks like in practice as part of the Ophelia process and the strengths and challenges of the specific methodologies employed. In the current paper, we have endeavoured to provide detail about the realities of conducting co-design workshops in this school context. This includes the preparation required, from developing relationships with the participating schools to logistical organisation of accessible resources. To the best of our knowledge, this co-design approach has not been demonstrated previously in a socioeconomically disadvantaged adolescent population in relation to health literacy. Of note, and in line with the Ophelia methodology, the use of a co-facilitator who was able to record, synthesise and reflect discussions (including a visual display) in real time with participants was found to be a valuable method of member checking the accuracy, collective agreement and authenticity of concepts. It also allowed participants themselves to make clear indication of the relative importance of concepts, minimising some of the potential researcher bias in the interpretation of these results. There were, however, some challenges encountered, particularly in analysing these findings, not least some of the deliberations as to the appropriateness of synthesising concepts across different school communities. In light of this, we acknowledge that while these findings may not be generalisable, some aspects of this study may be transferable to other contexts. In addition, it is simply not possible to adequately present and articulate the responses and engagement of all participants in the constraints of a journal article. Although concepts and selected quotes have been presented here (see Table 2), the wider insight provided by participants across the co-design workshops will inform the ongoing co-design of this intervention. The intervention will continue to be iteratively refined. Notably, this may include reference to COVID-19 (this stage of the project was conducted throughout the winter of 2019–2020, with Irish restrictions implemented in March) and sexual health, which, although a prevalent topic in the literature [64], was not dominant within the initial needs analysis stage of this project. In addition, we hope to include parents and guardians in future work as they represent a key stakeholder in adolescent health literacy and health behaviours [65] yet are considered “hard to reach” and are subsequently hardly reached [37]. Despite considerations and actions from the research team, the group format of these workshops may have been impacted by peer pressure, social desirability or the power dynamic and the addition of interviews in future stages of the project could be a way of accounting for this.

## 5. Conclusions

The current study identified health topics and capacity building actions that could be used to improve the health literacy and health outcomes of Irish adolescents attending designated socioeconomically disadvantaged schools. Five specific health areas (pre COVID-19) were identified: food choices, mental health and wellbeing, physical activity and sedentary behaviour, sleep and substance misuse. Participants within this study spoke of a number of social and environmental factors that influence health and of strategies and potential solutions that spanned across the whole school environment, curriculum and community. The current paper details the efforts to engage, and the importance and the benefits of engaging, with groups that are “hardly reached” from the earliest stages of a research project that concerns them. The co-design workshops placed young people and their communities at the centre of the research process and co-design procedures such as those detailed in this paper could (and we would advocate, should) be a core element of intervention design in the future. Such findings enable greater understanding of young people’s views and experiences, but, crucially, this study details methods that enabled young people and their school communities to voice these views and experiences. By working with members of the school community throughout the research process, it is hoped future intervention strategies can also be co-designed, implemented and iteratively evaluated with key stakeholders to generate transformative meaningful change.

## Figures and Tables

**Table 1:** Breakdown of study participants across schools.

School	Number of Students	Number of Staff
1	9	5
2	6	8
3	10	9
4	7	9
Total	32	31

**Table 2:** Health topics and capacity building actions identified by participants.

Health Topic	Capacity Building Actions	Cited in:		Example Quote
		Student Workshop	Staff Workshop	
Food Choices	Cooking classes	X	X	*“The parents love it (the provision of cooking classes). Sometimes it feels like we’re patronising parents, but that’s not how we want them to feel, and we have them come in (to school for the classes)”.* Staff Member *“We spend lots of time learning about the food pyramid, learning about health stuff, and then in our practical lessons we only cook stuff like muffins”.* Student
	Understanding food labels	X		*“So like the government is taking a little bit of charge now on all the back of food labels they have the amount of calories on them and the same on some menus now they must have it but there’s no law saying it has to be accurate”.* Student
	Policy changes around the sale of energy drinks to adolescents	X		*“Maybe you could put a restriction (on the sale of energy drinks), like how alcohol restriction is (sold)… 18”.* Student
	Student survey for food options in canteen	X		*“We have a (school) committee, and we’ll be changing what we have (on offer at lunchtime) to give other options”.* Student
Mental Health and Wellbeing	Advertise counsellor services better	X		*“I personally would love to see the stigma against mental health… it’s never going to be completely gotten rid of, but just work done and have people educated about it because in our school no one talks about it”.* Student
	Development of life skills (e.g., debating team)	X		*“We learn about like health…but we need the life benefits. Like, how to spend your money”.* Student
Physical Activity and Sedentary Behaviour	Non-competitive activities	X	X	*“I don’t do any sports, I have in the past but I don’t enjoy them but when I go with my friends I have realized that we actually walk a lot so we are keeping fit even though we are just hanging out with our friends”.* Student *“We need to try different sports in the school, or have other clubs, not even sports for those that might not be into that sort of thing”.* Staff Member
	Access to local sport facilities	X		*“I’m not sure if there’s a gym? Like a fitness gym we (the young people) can join…But they’re really popular…”.* Student
	Link with local sports groups	X	X	*“Swimming, hurling, football…”.* Student *“We could have more advertisements about clubs around the area. Announcement over the intercom or come into classes and tell students what’s going on”.* Staff Member
Sleep	Link with parents to share information (e.g., screen time applications)	X	X	*“Like my friend… he has one of the screen timers on his Xbox. He may be on it for an hour and then he has to go off”.* Student *“Some have that (ability to deal with influences on health) in their background in terms of social, family… you want to look at all of those units”.* Staff Member
Substance Misuse	External role models	X	X	*“That sounds like my cousin, he used to… he would smoke… but I’m not sure exactly what it was. He’s quite smart but he’s just lazy, he doesn’t have the motivation”.* Student *“They can actually see the experience of others who have succeeded, all the adversity and they were able to make good choices. The ones from 5th year or 6th year (older students), they can come in and show these guys “I did it”, they’ll (the older students will) be seen as superheroes and whatever. We need a person they can identify with”.* Staff Member
Generic Themes (across all health areas)	Education for both staff and students to show long term impact of health topics (especially smoking/vaping/drinking/drugs/gaming/lack of sleep)	X		*“Sometimes these students they know, but they don’t care. They don’t see it directly affecting them…*”. Staff Member
	Strengthen cross-curricular links		X	*“A short course (a Junior Cycle offering in Irish education) that actually tackles the issues that we’re explaining there (in response to the vignettes) in a way that actually makes it real for schools… yeah…. and provides the materials, provides resources, provides links with others and allows you (the teacher) to teach it…some of these issues that they had”.* Staff Member
	Partnerships with existing schemes	X	X	*“I’m not sure if there’s a gym? Like a fitness gym we can join…But they’re really popular…They’re already really good”.* Student *“It’s 3:40 p.m. when we finish. It’s a different world for us as teachers, we don’t know what goes on the streets for the kids”.* Staff Member
	Interactive, applied and relevant workshops	X	X	*“Most classes is just you come in and you sit down and get asked a couple of questions, then you go home and do homework …tons of homework”.* Student *“(There needs to be) …some practical hands on (activities)…. Take ownership of what they want”.* Staff Member
	Fun and engaging off-site delivery		X	*“(Learning about health need to be)… Anything else other than sitting down and just doing that (being talked at by a teacher), because most classes is just you come in and you sit down and get asked a couple of questions, then you go home and do homework…lots of homework”.* Student
	Target younger age groups (12–13-year-olds)	X	X	*“Well, she’s (the vignette character is) 15, this could be the beginning of the end for her now because she’s reaching teenage years now and starting to do… (more negative health behaviours) she could be on a slope now”.* Student *“I think the only way you’re going to change it, I think its first years. Forget about anything else, forget the rest. I think when they start coming into first years, they need a programme (about health and wellbeing to engage with in school)*”. Staff Member

## Data Availability

Data is available upon reasonable request.
